# The Influence of Parent Education on the Neurobehavior and Sucking Reflexes of Very Preterm Infants

**DOI:** 10.3390/brainsci12070840

**Published:** 2022-06-28

**Authors:** Roksana Malak, Katarzyna Wiecheć, Brittany Fechner, Tomasz Szczapa, Joanna Kasperkowicz, Maja Matthews-Kozanecka, Teresa Matthews Brzozowska, Oskar Komisarek, Włodzimierz Samborski, Ewa Mojs

**Affiliations:** 1 Department and Clinic of Rheumatology, Rehabilitation and Internal Medicine, Poznan University of Medical Sciences, 61-545 Poznan, Poland; blemus001@gmail.com (B.F.); joanna.monika.borek@gmail.com (J.K.); wlodzimierz.samborski@ump.edu.pl (W.S.); 2 Department of Clinical Psychology, Poznan University of Medical Sciences, 60-812 Poznan, Poland; k.wiechec@interia.eu (K.W.); ewa_mojs@poczta.onet.pl (E.M.); 3 Neonatal Biophysical Monitoring and Cardiopulmonary Therapies Research Unit, II Department of Neonatology, Poznan University of Medical Sciences, 60-535 Poznan, Poland; tszczapa@gmail.com; 4 Department of Social Sciences and the Humanities, Poznan University of Medical Sciences, 60-806 Poznan, Poland; pszczolka-maja@o2.pl; 5 The Chair and Clinic of Maxillofacial Orthopaedics and Orthodontics, Poznan University of Medical Sciences, 60-812 Poznan, Poland; teresamatbrzo@gmail.com (T.M.B.); oskarkomisarek@gmail.com (O.K.)

**Keywords:** neurobehavior, neonates, prematurity, sucking, NBAS, early rehabilitation, parent education

## Abstract

The diagnosis of neurobehavioral problems in very preterm neonates helps with planning and applying proper and direct therapeutic interventions. (1) Background: The aim of this study was to determine the direct impact of neurobehavior on the sucking reflex and eating abilities of neonates. (2) Methods: We assessed 18 preterm neonates twice hospitalized at the Gynecology and Obstetrics Clinical Hospital through the use of the Neonatal Behavioral Assessment Scale (NBAS). (3) Results: We found that that a neonate’s sucking ability positively correlated with the activity level item from the motor system cluster of the NBAS. (4) Conclusions: Neurobehavior should be closely assessed in very preterm neonates. Firstly, because assessments can detect fundamental problems and help a practitioner plan for early intervention. Secondly, the education of parents regarding the neurobehavior of their child can help in the facilitation of feeding skills and the planning of early rehabilitation.

## 1. Introduction

Prematurity may be a risk factor for the development of motor and cognitive impairments. According to the World Health Organization, 6–7% of children are diagnosed with a developmental disability [1]. The final trimester of pregnancy is especially crucial for brain development and any risk factors in utero, as prematurity may influence cerebral function. Assessments of neurological maturity, neurodevelopment, and neurobehavior as a foundation for complex neuromotor activity should include oral feeding behaviors [2].

Preterm infants are at a higher risk for developing problems with feeding [3]. Approximately 40% of premature infants between the ages of 25–37 weeks of gestation develop problems with eating [4]. Oral feeding is one of the most important issues for parents to address. This is why medical staff working in a NICU should facilitate parent–infant bonding by educating parents on how to effectively encourage communication, participate in neonatal care, and breastfeed [5]. There are different methods of providing education to parents, for instance, maternal participatory guidance sessions and telephone calls with a nurse-community advocate team. It has been shown in previous studies that intervening with both parents and their infant is a beneficial approach to helping premature neonates achieve the social interaction patterns important for optimal development [6]. Educating parents helps empower them by showing them how important they are for their children and that they are able to observe and take care of their preterm infants. Many parents often feel apprehension and may question their ability to care for their baby. Supporting parental involvement and providing education improves parental confidence and decreases anxiety [7].

Successful and efficient feeding creates opportunities for parents and their infant to have positive interactions [4]. This has a longitudinal effect on an infant’s emotional development, social learning, and overall health [8]. There are many aspects that may influence effective nutritive sucking. Feeding skills may be positively associated with motor development, while delayed sucking behaviors may reflect abnormal neurological functioning [9]. The relationship between motor development and oral feeding may be associated with behavior, language development, and cognitive ability. Although the assessment of some motor skills in preterm infants in the neonatal unit seems to be premature, there are some methods which are valid in the prognostication of the future development of an infant even if they are performed in the first eight weeks of life during hospitalization [9].

For instance, a practitioner could use the General Movement Assessment (GMA), Alberta Infant Motor Scale (AIMS), or the Neonatal Behavioral Assessment Scale (NBAS) to assess an infant in the first eight weeks of life [10]. The use of these assessments, especially with preterm infants, can enable a practitioner to recognize some of the earlier symptoms of potential neurodevelopmental impairments which could then provide them with the opportunity to improve neurodevelopmental outcomes through the use of early rehabilitation interventions [11]. Using the AIMS revealed 100% comparison among experts [12]. The GMA is a useful clinical instrument for the early identification of cerebral palsy and is a good predictor of later cognitive and behavior outcomes, even at school-age. The sensitivity of the GMA is 98%, so is comparable to a cranial ultrasound, magnetic resonance (MR), and a neurological examination in terms of its fidelity [13]. The GMA enables the practitioner to evaluate the effectiveness of therapeutic interventions [14]. Although the GMA may be used with preterm infants, some other factors may influence the motor performance of preterm infants, as many of these infants are considered medically fragile and may be unable to maintain sufficient energy reserves to produce the best results throughout the evaluation [15]. One factor is the autonomic nervous system (ANS). Autonomic maturation tends to be impaired in premature babies [16]. The specific patterns of an immature ANS in preterm infants may underlie a delay in development [17]. This is why the immaturity of the ANS could present a real danger to preterm infants [18].

According to the Synactive Theory of Development, an autonomic state is one of the five subsystems of neonatal behavioral organization. The other four subsystems required for proper development are: motoric, behavioral, interactional/attentional, and self-regulation [19]. The ANS is described as the first in the hierarchical order of the Synactive Theory of Development [20]. There is the presence of a synchronous relationship between the autonomic and motoric systems in preterm infants [19]. Another item is muscle tone assessment [15]. Early muscle hypotonia may be associated with feeding problems [21]. Additionally, the NBAS consists of items in reference to the ANS and general muscle tone [22]. The sensitivity of this scale is approximately 50–78%, with a specificity of 94–97%. Another scale that considers the motor system and the ANS system is the Assessment of Preterm Infants’ Behavior (APIB) [23]. Although the APIB has demonstrated strong concurrent validity with MRIs and electroencephalograms, a drawback is that it takes a lot of time to perform in a neonatal unit [24]. The autonomic system’s signals are evaluated based on the NBAS and APIB, since they are similar and have many items in common. The ANS is described by the presence of startles, tremors, and color fluctuations. In reference to the APIB, respiratory patterns and visceral responses (e.g., gagging, bowel movement strains, and actual defecation etc.) are also taken into consideration. This is why using more than one method of evaluation should be recommended [25].

Knowledge of ANS activity may assist in the monitoring of prematurity as well as various disease states, such as severe metabolic diseases and brainstem pathologies, in preterm infants. Even infants born prematurely and medically fragile display reliably observable behaviors along the lines of systems of the body, such as the ANS and other systems as well. Therefore, this is the reason why those systems should be assessed. In general, there is still not enough known about the relationship between the ANS and early social human behavior [26]. However, there is more and more research about it. For instance, Christina Koumarela et al. (2021) investigated the regulatory role of the ANS and social reciprocity in preterm infants which may have big implications for the fields of medicine and psychology [26]. Another study showed that there are some methods which may help with ANS regulation. For instance, facilitating early interactions and an emotional connection between preterm infants and their mothers may effectively optimize development in preterm infants. Furthermore, repeated mother–infant calming interactions positively influences on autonomic state and prosocial behaviors [27]. Knowledge about the ANS, as a part of neurobehavior, can help in supporting parents in identifying and responding to early communication and oral readiness signs which could then increase parental confidence in infant management and enable quicker discharges home. We would like to determine if there is any relationship between the motor system and ANS as well as in the development of feeding skills in preterm neonates.

Providing parental education about the condition of the child, including neurobehavior and feeding (sucking) in preterm infants, appears to have many benefits. One of them is reducing the number of hospitalization days in the neonatal unit [28]. Although sucking and swallowing is particularly vital for the early development of an infant, applying sucking therapy is not necessarily efficient in helping children with feeding problems [4]. This is why we would like to check whether supporting neurobehavior, including feeding and the education of parents in the ways of facilitating the neuromotor development of a child, is beneficial to a preterm infant. We hypothesized that providing parental education would be beneficial for the motor development of a child and for developing in infants the ability for them to be fed orally instead of via a tube. We were interested in the effects of parent education on infant neurobehavior and suspected that assessing some neurobehavior items would be beneficial. We also wanted to observe the factors associated with the feeding skills of a child.

## 2. Materials and Methods

### 2.1. Characteristic of the Research Group

We assessed 18 preterm neonates hospitalized at the Gynecology and Obstetrics Clinical Hospital. All parents of the 18 infants were of West Slavic Caucasian descent. Thirteen parents lived in towns and five in villages. In regard to the education of parents, nine mothers had a Master’s degree, four had vocational school education, and five had secondary general education. Six fathers had a Master’s degree, eight had a technical high school education, and four had a secondary general education. All parents worked and no one was unemployed. All neonates presented with feeding problems. Inclusion criteria included: a week of gestation before week 32 and a birth weight lower than 1500 g. The exclusion criteria included oxygen desaturation (SpO2 < 88%), a heart rate lower than 100 or higher than 205 beats per minute, active inflammation, sepsis, bone replacement, tumors, encephalopathy, hypotension, and lethal birth defects (e.g., Edwards, Patau etc.).

The average age of gestation was 29 ± 2 weeks (minimum 25 weeks postmenstrual age and maximum 32 weeks postmenstrual age). The average gestational age of the infants during the time of the NBAS assessment was 34 + 2 postmenstrual age. The average birth weight was 1230 g ± 229 g. Each parent was educated on how to appropriately engage in the following care strategies with their infant: observe, position for feeding, hold, play with, communicate with, care for, breastfeed, and use a pacifier. Additionally, each parent was taught how to maneuver and console their infant using the following sequence: lowering the voice, placing a hand on the infant’s belly, holding the infant’s arms, picking up the infant, rocking the infant, swaddling the infant, and finally applying a pacifier if needed.

### 2.2. Methods

We assessed each infant twice in the span of three months through the use of the NBAS [29]. We scheduled the first assessment at about 34 weeks of gestation when the coordination of sucking and swallowing should first appear. The second examination took place for each infant at a corrected age of three months, since corrected age is known as a reliable predictor of gross motor maturation or walking skills at 15 months of age [30,31,32]. We avoided any attrition or absence of participants by assessing an infant only if there was cohesion in opinion in its stable condition by a nurse or neonatologist. The qualified clinician, trained by the NBAS course provided by the Cambridge Brazelton Centre in the United Kingdom, administered as many as 28 behavioral items, 18 reflex items, and 7 supplementary items [33]. The NBAS takes into consideration clusters, such as: Habituation, social interaction, motor system, state regulation and organization, ANS, reflexes, and supplementary items [29]. We used the NBAS to check the neonates’ neurobehavior, including sucking behavior, as well as their ANS [29]. The Motor System cluster of the NBAS helped us to observe the motor development of neonates [34]. In regard to the Motor System cluster from the NBAS we considered: General Tone, Motor Maturity, Pull-to-Sit, Defensive, and Activity Level. We analyzed items both as a cluster (Motor Cluster) but also separately. In general, when assessing general tone via the NBAS, the best performance of a child was a score of 5, in which the tone was considered average, and the child laid with a relaxed tone. The higher the muscle tone a child presented, the more points the child received on the NBAS. The more flaccid the muscle tone, the less points a child received. Similarly, for Activity Level, a score of 5 meant moderate activity, while more points indicated that the child moved continuously and less than 5 points meant that a child’s activity was too low. Other items were based on a 0–9 scale where a higher number of points indicated better outcomes: smoother movements; better head, limbs and trunk reaction in the pull-to-sit item; and a more mature defensive reaction. We also checked sucking ability and gave two points to a child if they had a rhythmic suck, a score of 1 if they had a weak suck, or a score of 0 if no sucking reflex was present.

### 2.3. Analytic Strategy

Statistical analysis was performed using Statistica Version 13 software (TIBCO Software, Tulsa, OK, USA). The correlation between samples was measured using Spearman’s rank correlation coefficient. A *p*-value of <0.05 was considered statistically significant. A non-parametric Mann–Whitney U test was used to test for group differences in continuous variables. We also used McNemar’s test. This study was approved by the Bioethics Committee, consent ref. No. 481/21.

## 3. Results

The results from our study are presented in the tables below. Firstly, we present the results of NBAS motor cluster and ANS cluster in the Table 1.

In Table 2, we found that the motor system cluster and ANS cluster changed after providing education to parents. More significant changes were seen in motor maturity, defensive reaction, and in the motor system cluster in general. In reference to the ANS cluster, bigger changes were present in liability of skin color (*p* = 0.033).

We showed that sucking ability was positively correlated with motor development, as measured by the NBAS Motor system cluster, and was especially statistically significant with the activity level item (Table 3). However, if a child was overreactive which was shown in the activity level item (*p* = 0.012), it disturbed the process of sucking and feeding (r = −0.55).

We also found a positive correlation between pH value and sucking behavior (r = 0.534, *p* = 0.49). The higher the pH value, the better the sucking and feeding a child presented.

Early intervention, which included parental education such as on appropriately positioning an infant, helped him or her in gaining the ability to eat without any special equipment, such as a feeding tube. We found that 100% of our participants did not have to be fed via a tube following our intervention of providing parental education on how to position a child, which pacifier on a bottle to use, or how to successfully facilitate breastfeeding (Table 4). These results are significant, since in the first assessment only four children were orally fed, while 12 children were fed via a tube. In our second assessment, 16 children were orally fed (either via breast or bottle).

## 4. Discussion

Sucking seems to be the most fundamental motor skill in a newborn’s movement repertoire. The assessment of motor development and sucking should be combined. Additionally, the facilitation of motor development may have an impact on sucking and vice versa. Appropriate posture provides a stable base for facilitating facial actions such as feeding [29,34,35,36]. In particular, Craig et al. (2000) showed the correlation between the degree of muscle hypotonia, the level of development, and sucking. This strong relationship lasts until a child is seven months of age. The author of this study wrote that future research could look at the control of sucking and its correspondence with scores for spontaneous general movements as well as neurobehavior [37]. This is why we arranged our study to take into consideration these aspects.

Although, it is found that in high-risk neonates, the coordination of the suck–swallow–breathe sequence can be found as early as 34 weeks, we wanted to check what factor made the difference in the ability to suck earlier in these preterm neonates. Some studies have shown that infants were ready to feed earlier from a bottle [35]. The preterm infants who sucked irregularly were found to have delays in their motor development [37]. We observed the correlation between sucking behavior and motor development not only in the first examination but we also revealed that supporting motor skills and sucking enabled the infants to be fed without the use of a feeding tube, without any other additional equipment, and without any problem in the second examination of our research (Table 1 and Table 2). All participants in the study received their treatment and education from specialists (i.e., psychologist, physical therapist, speech therapist, orthodontist, neonatologist, and family assistant) in a neonatal unit and later at home. All the infants from the research group did not have any difficulties eating three months later.

Feeding may be treated as the primary regulator of a physiological state. Firstly, sucking requires the coordination of the striated muscles of the face with visceral changes in heart rate and breathing. The feeding challenge paradigm provides an opportunity for newborns and preterm infants to evaluate the status of coordinated physiological–behavioral sequences that require vagal regulation and the control of the striated muscles of the face, head, and neck. Porges (2011) monitored the integration of sucking behaviors with heart rate and respiratory sinus arrhythmia (RSA—the state of the vagal brake quantified as the amplitude of a periodic component in the beat-to-beat heart rate pattern) as infants sucked [38]. This is why we revealed that there is a correlation between sucking, motor system behavior, and the ANS. Therefore, we conducted our therapy taking into account not only sucking behavior but also the regulation of muscle tone in the face, head, and neck muscles and trying to make a child stable in regard to the ANS by applying appropriate positioning and education for parents on how preterm infants can be regulated [38]. The parent education we provided included the semi-elevated side-lying (ESL) position. The ESL position better supports the regulation of breathing during feeding, which allows very preterm neonates to maintain better physiological stability during feeding [39]. Burcu Aykanat Girgin et al. (2017) in particular found that the ESL position was more effective than the semi-elevated supine (ESU) position during feeding routines with preterm infants [40]. The physical therapist and physiologist encouraged parents to play with their children in a prone position, because a limited time spent in this position appears to cause developmental delays in infants [41,42]. In general, time spent in a variety of positions is important for infant motor development [43]. Monitoring early positioning practices is important because these may help in feeding [39]. Furthermore, Şadiye Dur and Duygu Gözen (2020) found that when care providers provided pacifiers to preterm infants and utilized cue-based feeding, feeding performance improved [44]. Suzanne Thoyre et al. (2013) also found that cue-based feeding should be assessed when analyzing effective feeding practices for preterm neonates in order to attain the best outcomes [45].

The relationship between the ANS and motor function has previously been shown [46]. We also revealed that the higher the motor maturity of a preterm infant, the lower the ANS cluster score they received. Abnormal ANS development has been linked to worsened neurodevelopment (lower score of motor development) outcomes in many studies [46,47,48,49]. This is why taking care of general health, considering environmental factors, and using appropriate positioning to regulate the ANS can help in supporting the neurodevelopment of a child [49]. Parental education should take into consideration the surrounding environment, such as excessive noise and body position, especially during the first six months of the postnatal period, since this time is fundamental for the maturation and development of the ANS and neurodevelopment [49]. According to Gomes et al. (2019), “Stress provoked by the surrounding environment leads to an increase in energy expenditure in premature newborns, which exerts a negative impact on neurological integration as well as growth and development” [49]. Even the response of neonates to painful procedures and the signs of ANS activation (sympathetic) change when children are placed into more comfortable positions [49].

Another aspect that plays an important role in the development of a child is umbilical cord arterial pH. We presented the relationship between sucking behaviors, umbilical cord arterial pH, and motor development in our previous study, as other researchers have also done [50]. A low umbilical cord arterial pH value may lead to hypoxia or acidosis which may then lead to intraventricular hemorrhage (IVH), cystic periventricular leukomalacia, and neurodevelopmental disorders. Therefore, children who have a low umbilical cord arterial pH value, especially lower than seven, should receive early intervention and receive special care from the very first moments of life [51].

In summary, we would like to show that the sucking reflex is an essential item of oral feeding that should not be assessed separately, but in accordance with motor development. The correlation between feeding and the motor system as well as the ANS is supported by the Synactive Theory of Development, as developed by Heidelise Als. The regulation and the homeostasis of a preterm neonate is dependent on the collaboration of the following subsystems: Autonomic, motor, behavioral, attention-interaction system and self-regulation system [52]. Even if a neonate is born preterm, there is still an internal need to self-regulate. The importance of incorporating the infant’s self-regulation by clinicians should be an aim in neonatal units [53]. The Synactive Theory suggests that caregivers of preterm neonates can assist infants in meeting self-regulatory goals by providing care according to the infant’s own behaviors [53]. Clinicians may therefore benefit from Als’ Synactive Theory when designing interventions for very young infants which take into account autonomic, motoric, state regulation, interactional/attentional and self-regulatory factors.

## 5. Conclusions

Neurobehavior should be observed and monitored in very preterm neonates. Firstly, because it can indicate any preliminary problems and help a practitioner plan for early intervention. Secondly, taking into consideration neurobehavior items may help with the facilitation of feeding skills and planning for the early rehabilitation of this vulnerable population.

## Figures and Tables

**Table 1:** Motor system cluster and ANS cluster from the NBAS.

	n	Average Value	Standard Deviation	Minimum	Maximum
General tone	16	4.87	2.475	1	9
Motor maturity	16	4.20	3.234	0	9
Pull-to-sit	16	4.40	4.188	0	9
Defensive	16	2.93	3.218	0	9
Activity level	16	3.40	2.849	1	9
Sum of Motor system cluster	16	19.80	1.252	2	45
Autonomic system: Tremulousness	16	1.38	0.806	1	4

**Table 2:** Comparison of the first and second examination.

	Number of Children		Average Value		Standard Deviation		Minimum		Maximum		*p*
Motor system NBAS	I exam	II exam	I exam	II exam	I exam	II exam	I exam	II exam	I exam	II exam	
General tone	16	16	4.87	5.75	2.41	1.18	1	4	9	9	0.284
Motor maturity	16	16	4.2	6.5	3.26	1.93	0	2	9	9	0.030
Pull to sit	16	16	4.40	6.44	4.21	2.15	0	2	9	9	0.101
Defensive	16	16	2.93	6.31	3.12	2.46	0	0	9	9	0.003
Activity level	16	16	3.40	7.00	2.78	2.22	1	2	9	9	0.012
Sum of Motor system cluster	16	16	19.80	32	11.25	7.09	2	12	45	41	0.005
Autonomic system NBAS											
Tremulousness	16	16	1.38	2.06	0.806	2.72	1	1	4	9	0.596
Startles	16	16	1.44	1.25	1.094	0.683	1	1	5	3	0.18
Lability of skin color	16	16	3.31	1.94	2.243	1.569	1	1	7	5	0.033
Sum of Autonomic System Cluster	16	16	6.125	5.25	2.305	3.43	3	3	10	13	0.279

A *p*-value less than 0.05 is statistically significant.

**Table 3:** The relationship between the NBAS Motor System cluster and sucking.

	General Tone	Motor Maturity	Pull to Sit	Defensive	Activity Level
r	−0.335	0.81	0.236	0.459	−0.550
*p*	0.222	0.774	0.398	0.085	0.034
N	15	15	15	15	15

A *p*-value less than 0.05 is statistically significant.

**Table 4:** The ability of oral feeding before and after parental education and early intervention.

	N	Average	Standard Deviation	Minimum	Maximum	*p*
Oral vs. tube feeding first exam	16	1.27	4.58	1	2	0.001
Oral vs. tube feeding second exam	16	2.00	0.000	2	2	

A *p*-value less than 0.05 is statistically significant, McNemar’s Test.

## Data Availability

The data that support the findings of this study are available per request from the corresponding author [R.M.].
